# Identification of platinum resistance-related gene signature for prognosis and immune analysis in bladder cancer

**DOI:** 10.3389/fgene.2023.1062060

**Published:** 2023-01-26

**Authors:** Sheng Li, Ming Jiang, Lin Yang, Fucun Zheng, Jiahao Liu, Xiong Situ, Xiaoqiang Liu, Liu Weipeng, Bin Fu

**Affiliations:** ^1^ Department of Urology, Nanchang, China; ^2^ The First Affiliated Hospital of Nanchang University, Nanchang, China

**Keywords:** bladder cancer, platinum resistance, long non-coding RNA, os, prognosis model

## Abstract

**Purpose:** Currently, there is limited knowledge about platinum resistance-related long non-coding RNAs (lncRNAs) in bladder cancer. We aim to identify platinum resistance-related lncRNAs and construct a risk model for accurate prognostic prediction of bladder cancer.

**Methods:** Transcriptomic and clinical data were extracted from The Cancer Genome Atlas (TCGA) database, and platinum resistance-related genes were obtained from HGSOC-Platinum. The platinum resistance-related lncRNAs were obtained by the Spearman correlation analysis. Then, we constructed a risk score model through Cox regression analysis and the LASSO algorithm. The model was verified by analyzing the median risk score, Kaplan-Meier curve, receiver operating characteristic (ROC) curve, and heatmap. We also developed a nomogram and examined the relationship between the risk score model, immune landscape, and drug sensitivity. Lastly, we assessed the differential expression of PRR-lncRNAs in the cisplatin-resistant bladder cancer cell line and the normal bladder cancer cell line using qRT-PCR.

**Results:** We developed and validated an eight-platinum resistance-related lncRNA risk model for bladder cancer. The risk model showed independent prognostic significance in univariate and multivariate Cox analyses. Based on multivariate analysis, we developed a nomogram. The modified model is both good predictive and clinically relevant after evaluation. Furthermore, immune-related and drug-sensitivity analyses also showed significant differential expression between high and low-risk groups. The qRT-PCR demonstrated that most of the lncRNAs were upregulated in cisplatin-resistance cancerous tissues than in control tissues.

**Conclusion:** We have developed a predictive model based on eight platinum resistance-related lncRNAs, which could add meaningful information to clinical decision-making.

## Introduction

Bladder cancer (BLCA) is the world’s 10th most commonly diagnosed cancer (Babjuk et al., 2022). Although nearly 75% of bladder cancers are non-muscle-invasive (NMIBC), 45%–50% of patients with NMIBC will experience recurrence, and 6%–40% will progress (Slovacek et al., 2021). Patients with NMIBC are prone to develop muscle-invasive bladder cancer (MIBC) after repetition and have a high risk of metastasis and poor prognosis, with a few surviving for more than 5 years (Chou et al., 2016; Malmstrom et al., 2017). Approximately 50% of MIBC patients eventually develop the disease at distant sites because of disseminated micrometastases, even after undergoing radical cystectomy and pelvic lymph node dissection (Patel et al., 2020). Hence, identifying specific tumor factors and providing new biomarkers are necessary to accurately diagnose, treat, and predict bladder cancer’s outcome.

Platinum-based chemotherapy drugs are one of the most commonly used drugs for treating various tumors, especially for the systemic management of muscle-invasive and advanced bladder cancer (Ghosh, 2019). Initially, sensitive tumors, frequently observed in cancers, eventually develop chemoresistance. Unfortunately, the development of platinum resistance results in significant tumor recurrence and decreased overall patient survival (Hu et al., 2018). Non-coding RNAs that are longer than 200 nucleotides are long non-coding RNAs (LncRNAs). Since the development of high-throughput sequencing in recent years, many non-coding genes have been discovered to regulate the occurrence, development, metastasis, and chemotherapy resistance in cancers (Gao et al., 2020; Liu et al., 2020; Wu et al., 2020; Li et al., 2021a; Lu et al., 2021). It also significantly impacts bladder cancer, such as lncRNA KCNQ1OT1 facilitates the progression by targeting MiR-218–5p/HS3ST3B1 (Li et al., 2021b), and lncRNA CASC11 promotes cancer cell proliferation in bladder cancer through miRNA-150 (Luo et al., 2019).

Nevertheless, the role and prognostic value of platinum resistance-related (PRR) lncRNAs in BLCA have yet to be expounded. Consequently, we investigated the correlation between bladder cancer and PRR lncRNAs. As well as functional enrichment analysis of PRR lncRNAs, we analyzed immune cell infiltration, immune checkpoints, tumor mutational burden (TMB), immunotherapy, and drug sensitivity between high- and low-risk patients. Besides, we used a nomogram to visualize the overall survival of BLCA patients. It is hoped that new biomarkers can be provided for the personalized treatment of BLCA patients.

## Methods

### Data download and processing

The Cancer Genome Atlas (TCGA) database was accessed to obtain RNA sequencing data, tumor mutational burden (TMB) data, and related clinical information on bladder cancer patients. Transcriptome FPKM data was extracted using Strawberry Perl for further analysis. Genes expressing less than one in more than half of the samples were deleted. Moreover, clinically incomplete samples were excluded from the follow-up clinical correlation analysis. The results of comprehensive immunogenomic analyses of bladder cancer were obtained from The Cancer Immunome Database (TCIA, https://www.tcia.at/home). Platinum resistance-related genes were downloaded from HGSOC-Platinum (http://ptrc-ddr.cptac-data-view.org). Using the limma package in R software, a differential expression matrix for platinum resistance-related genes (PRR) was created. The criteria for differential expression analysis were | log 2 (fold change) | >1 and a false discovery rate (FDR) < 0.01.

### Identification of platinum resistance-related (PRR) LncRNAs

Spearman correlation coefficients were calculated based on differential expression PRR genes and lncRNA expression profiles to recognize platinum resistance-related lncRNAs (|R2 | >0.45 and *p* < 0.05).

### Construction of platinum resistance-related prognostic signature and GSEA

Firstly, univariate Cox regression analysis was utilized to evaluate the prognostic value of PRR lncRNAs. When the *p*-value was lower than 0.01, it was incorporated into the LASSO regression analysis. Then, based on the above results, we developed the platinum resistance-related prognostic model. Platinum resistance-related prognostic scores for each patient were calculated as follows: Risk score = (Coef (lncRNA1) * expression lncRNA1) + (Coef (lncRNA2) * expression lncRNA2) +……+ (Coef (lncRNA n) * expression lncRNA n). Eventually, due to the median risk score, patients were divided into low- and high-risk groups. The Kaplan–Meier curve was generated with the log-rank test to compare the two groups’ overall survival (OS). To evaluate the predictive performance of the signature, we used the ‘timeROC’ R package to generate a receiver operating characteristic curve (ROC). A heat map was used to show the difference in platinum resistance-related lncRNA expression profiles between the high/low-risk groups. We randomly split the entire cohort into a 1:1 train and a test set for internal validation to assess the risk model feasibility. Validation cohorts were calculated using the same formula as the total cohort, and the same validation method was applied. We used the Gene Set Enrichment Analysis (GSEA) to examine the molecular mechanisms underlying low- and high-risk groups. *p* values less than 0.05 were considered statistically significant.

### Building and validating a nomogram

Univariate Cox and multivariate Cox regression analyses were used to identifying potential prognostic factors for the risk model and clinical features. Then, we constructed a nomogram by incorporating the meaningful variables (*p*< 0.05). Clinicians can easily use the nomogram to assess 1-, 3-, and 5 year overall survival in bladder cancer patients. The receiver operating characteristic (ROC) and calibration plots were calculated to estimate the discriminative accuracy of the nomogram. All of these will be validated on training and test sets.

### Comprehensive analysis of the relationship between the risk model and tumor microenvironment and immunity

The ESTIMATE algorithm was used to assess immune infiltration in bladder cancer patients (Supplementary Table S1). The difference in immune cell infiltration between the high-risk and low-risk groups of patients was evaluated using TIMER, CIBERSORT, CIBERSORT-ABS, QUANTISEQ, MCP-counter, XCELL, and EPIC algorithms. In addition, the potential immune checkpoint was acquired from previous literature. We detected the expression levels of immune checkpoint-related genes between the two groups. Furthermore, we used TCIA data to predict the relationship between platinum resistance-related prognostic scores and immunotherapy sensitivity. TMB between the two groups was also analyzed. Box plots were generated to visualize the differences.

### Drug sensitivity analysis

We use the “pRRophetic” package in R software to predict the drug’s half-maximal inhibitory concentration (IC50) value between the high-risk and low-risk groups. Moreover, we considered *p* values less than 0.05 to be statistically significant. Box plots were generated to visualize the differences.

### Cell culture and qRT-PCR

Human BC cells of T24 were purchased from the Cell Bank of Culture Collection of the Chinese Academy of Sciences, Shanghai Institute of Cell Biology (Shanghai, China). The exponential growth phase T24 cells were selected, and 200 μg/mL was chosen as the initial drug concentration according to the pre-experiment. The same concentration was repeated three times, each 2 days. Continue using the previous concentration of cisplatin for 2 days after passage, and then gradually increase the concentration. If the cell condition is not good, replace the medium without cisplatin. When the cell condition is normal, continue to add medicine. A cisplatin-resistant bladder cancer cell line, T24-CDDP, was established after cisplatin continued for 10 months. The T24-CDDP cell lines were validated by Cell Counting Kit-8 (CCK-8) assay, and GraphPad Prism9 was used to plot the cell IC50. All cells are cultured in Dulbecco’s modified Eagle’s medium (DMEM; Gibco) and at 37°C in 5% CO2. Invitrogen TRIzol reagent was used for total RNA extraction and the Takara PrimeScript RT reagent Kit for cDNA synthesis. Real-time quantitative PCR was performed using SYBR Green (Roche, Switzerland). Glyceraldehyde 3-phosphate dehydrogenase (GAPDH) was used as an endogenous reference. At least three replicates of each reaction were performed. Supplementary Table S2 shows the primer sequences.

## Result

### Basic information

**FIGURE 1:**
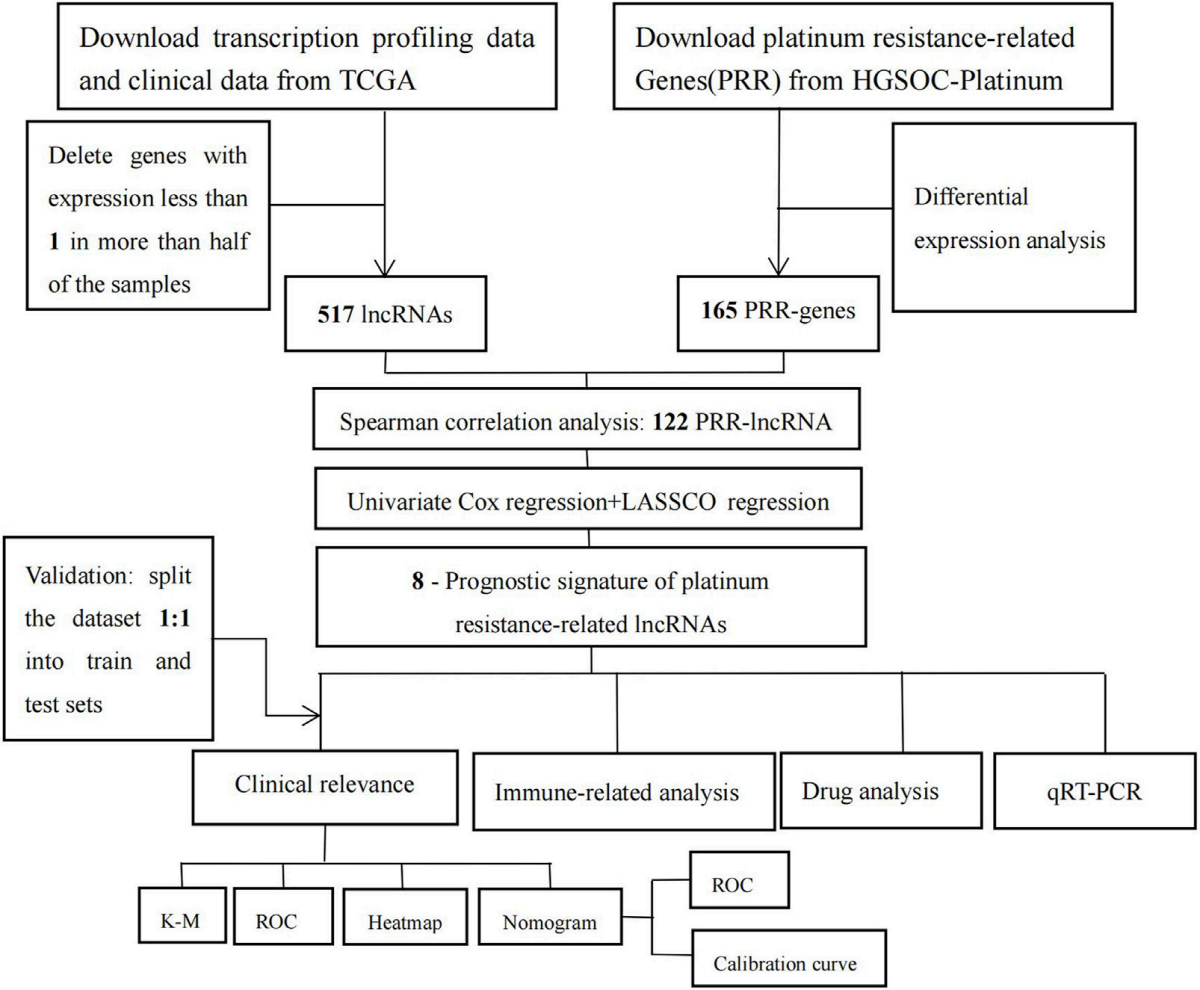
Flow-process diagram of the study.

Figure 1 shows the flowchart of our study. We obtained gene expression profiles of 431 bladder tumor patient samples, including 412 tumors and 19 adjacent normals, from the TCGA database. Samples with incomplete clinical information were removed. Supplementary Table S3 contains the clinical data for the remaining 372 tumor samples. Then we randomly split the entire cohort into a 1:1 train and a test set for internal validation. Data on 412 bladder cancers containing information on immunotherapy were downloaded from the TCIA database (Supplementary Table S4).

### Differentially expressed (DE) platinum resistance genes and LncRNA

Supplementary Table S5 records that 936 platinum resistance genes were extracted from HGSOC-Platinum. A comparison of bladder cancer tissues with normal tissues identified 165 DE genes (78 were upregulated and 87 were downregulated). Supplementary Figure S1 shows the heatmap evaluation of DE genes. In Supplementary Figure S2, a volcano map represents the distribution of all DE genes according to log10FDR and log2FC. Throughout the gene expression profiles, 16882 lncRNAs were identified. Five hundred eleven lncRNAs remained after deleting genes with an expression of less than one in more than half of the samples (Supplementary Table S6). Then, we identified 122 platinum resistance-related lncRNAs by correlation Spearson analysis, as shown in Supplementary Table S7.

### Construction and validation of a platinum resistance-related lncRNA risk model

**FIGURE 2:**
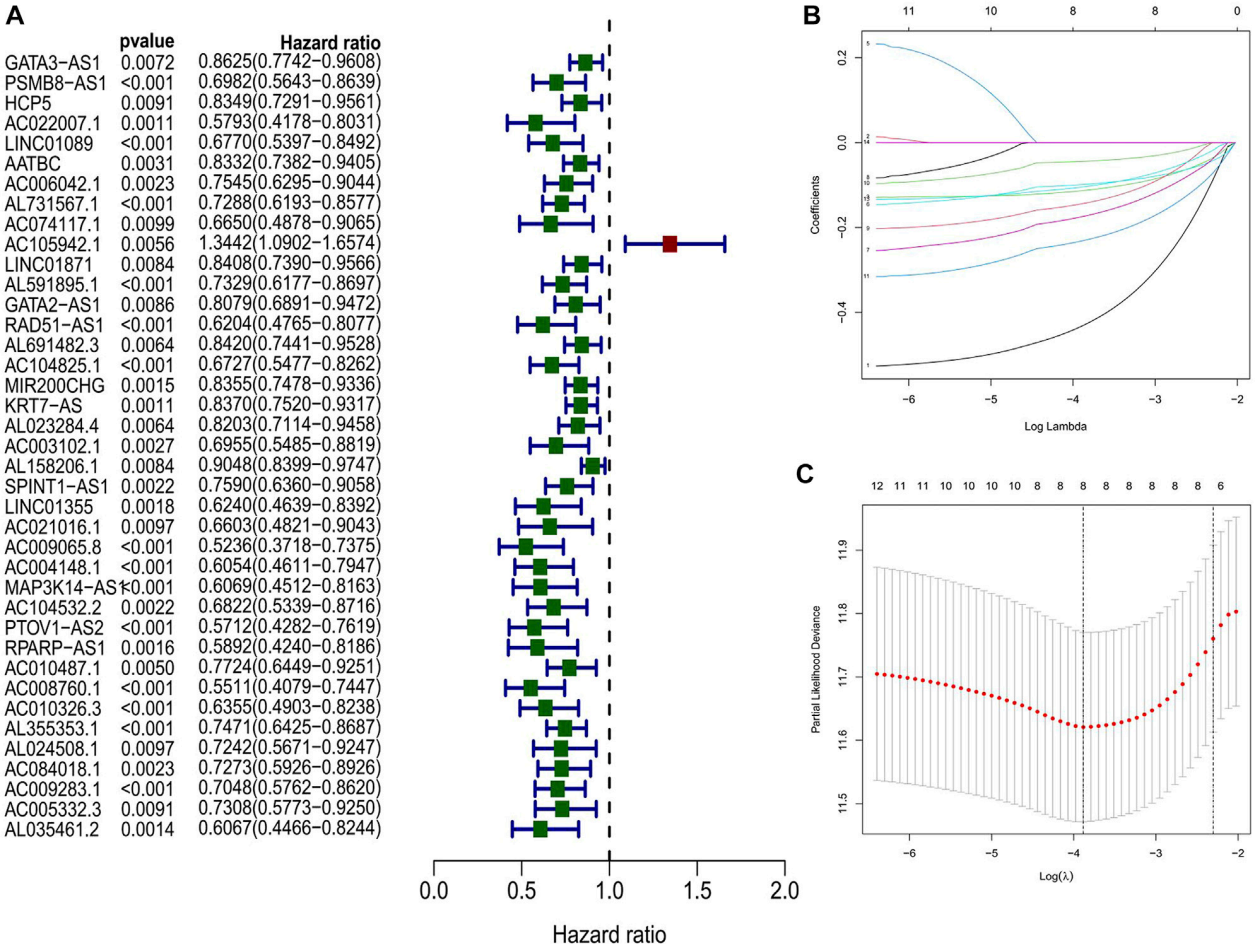
**(A)** Identification of prognostic PRR by univariate Cox regression analysis in the whole group **(B, C)** Lasso regression analysis in the entire group.

**FIGURE 3:**
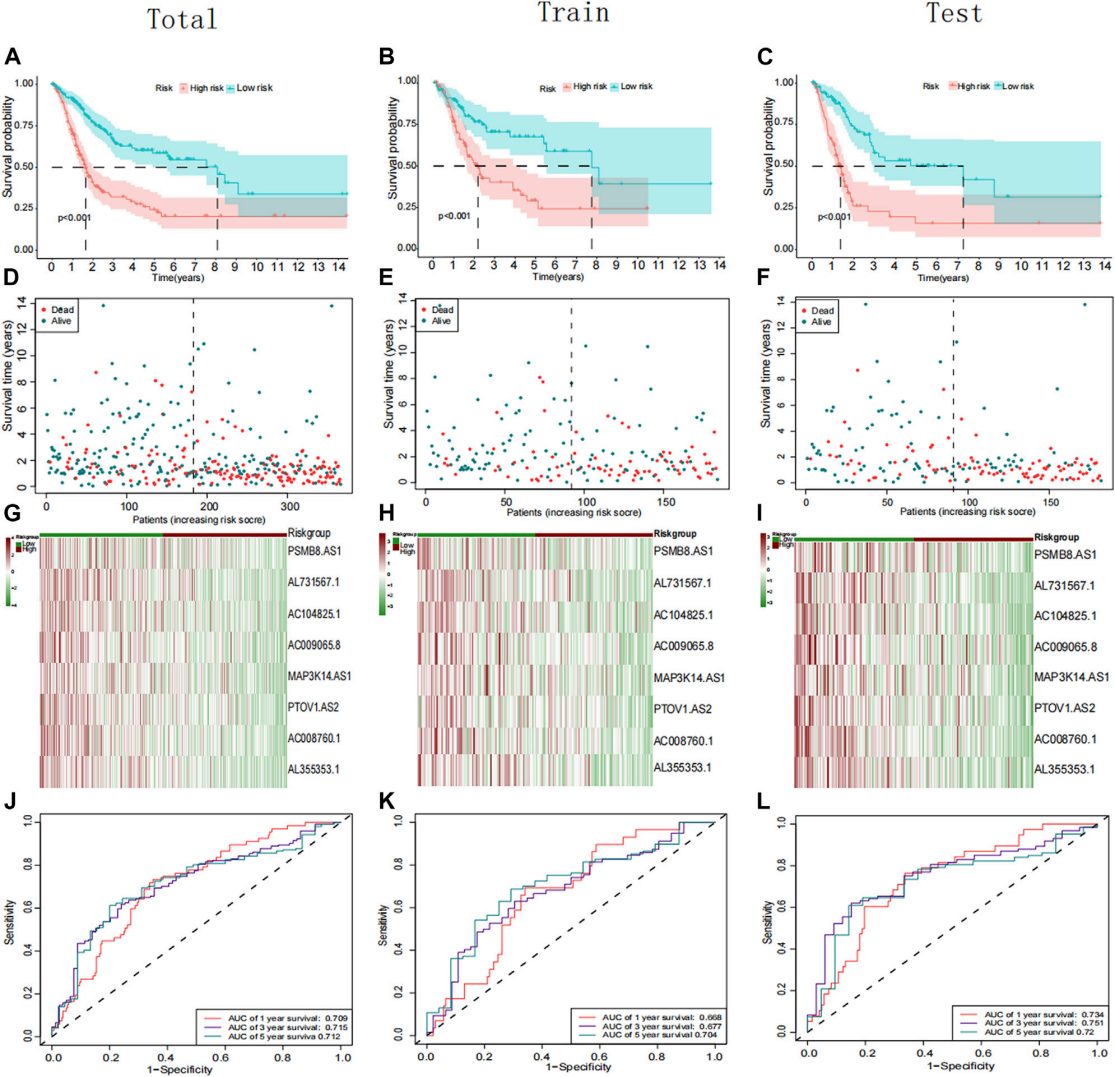
Prognostic analysis of the PRR lncRNAs signature in the total, training cohort, and testing group. **(A–C)** Kaplan–Meier curve of the patient in the whole, training cohort, and testing groups. **(D–F)** The rank of calculated risk scores in the total, training cohort, and testing groups. **(G–I)** Heatmap showed the differences of 8 PRR lncRNAs in the whole, training cohort, and testing groups. **(J–L)** Time-independent receiver operating characteristic (ROC) analysis in the total, training cohort, and testing groups.

Using univariate Cox regression, 39 platinum resistance-associated lncRNAs were identified. Other than AC105942.1, which was a high-risk prognostic LncRNA, all others were low-risk (*p* < 0.01, Figure 2A). In the LASSO regression analysis, eight platinum resistance-related lncRNAs were associated with prognostic factors in bladder cancer (BCa) patients (Supplementary Table S8). It was verified through cross-validation that the LASSO regression analysis optimal value was the right one (Figures 2B, C). The formula of the risk score was as follows: Risk score = (−0.431424975893598*PSMB8-AS1) + (−0.1130343821 6125*AL731567.1) + (−0.0984074363105057* AC104825.1) + (−0.173932427517578*AC009065.8) + (−0.1434206 81656449* MAP3K14-AS1) + (−0.0447654488946425*PTOV1-AS2) + (−0.231285006432146*AC008760.1) + (−0.10488485275485*AL35 5353.1). This risk model divided patients into high-risk and low-risk groups based on the median risk score. The Kaplan-Meier survival analysis showed that low-risk BCa patients had a significantly better overall survival than patients at high risk (Figure 3A). Moreover, based on the risk model, the scatterplot demonstrated a correlation between survival time and risk score for BCa patients. There was a correlation between patients’ risk scores and their mortality from bladder cancer. The higher the score, the greater the risk (Figure 3D). As shown in the heat map (Figure 3G), these eight-platinum resistance-related lncRNAs were highly expressed as protective factors in the low-risk group. Lastly, overall survival AUCs of 1-, 3-, and 5 years were 0.709, 0.715, and 0.712, respectively (Figure 3J). In the training and testing groups, we validated the risk model. These two groups used the same methods to identify high-risk and low-risk patients. Figure 3B and Figure 3C illustrate the relationship between risk scores and survival. The prognostics between the different risk patients in the training and testing groups were shown in Figure 3E and Figure 3F. There was a significant decrease in the overall survival of the high-risk group compared with the low-risk group. The heat maps were consistent across the entire group (Figures 3H, I). Figures 3K, L showed that both training and testing groups achieved ideal AUC values.

### Construction and assessment, a new type of nomogram

**FIGURE 4:**
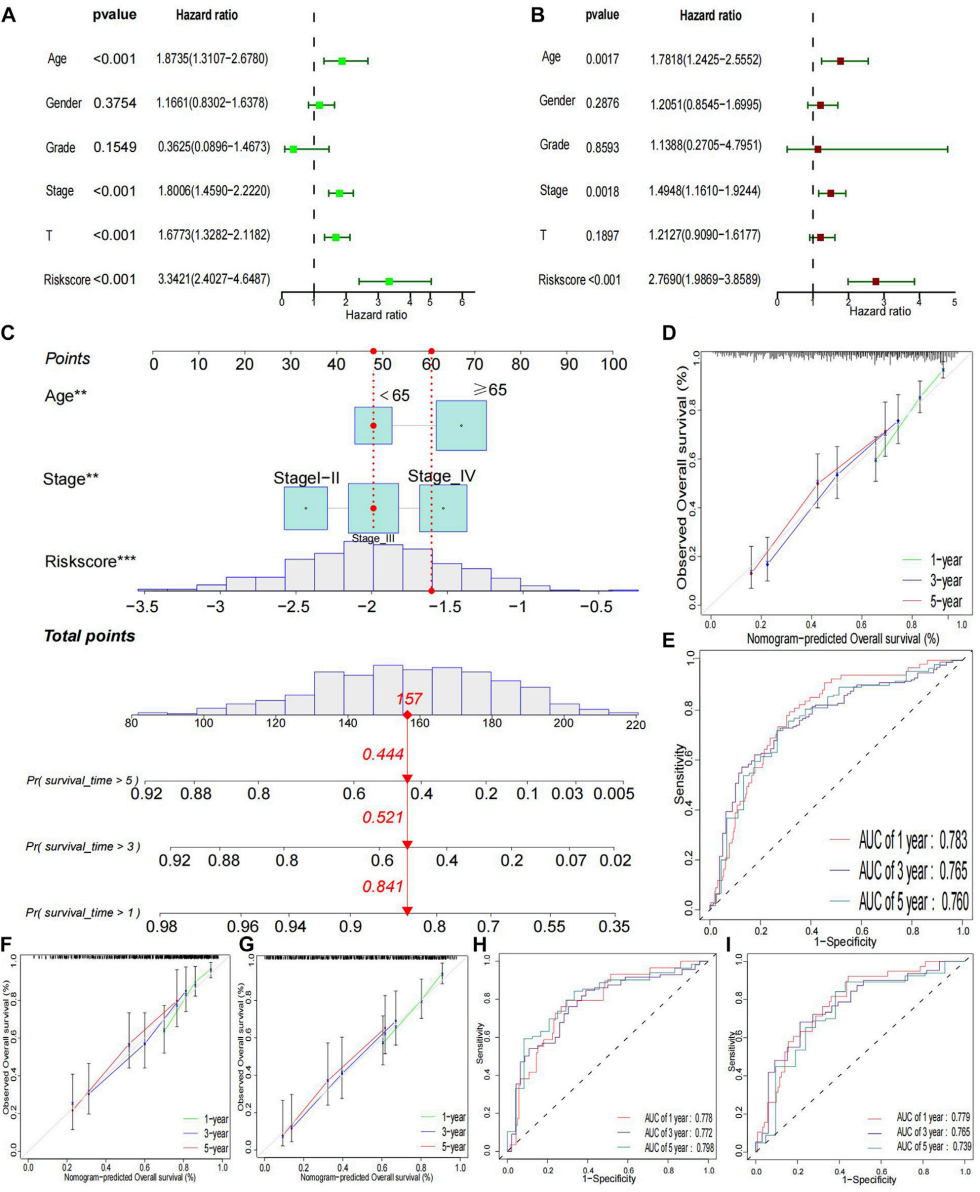
Clinical relevance Analysis and Validation **(A)** Univariate prognostic analysis. **(B)** Multivariate prognostic analysis. **(C)** Constructed a nomogram in whole groups. **(D, E)** Calibration curves and ROC for 1-, 3-, and 5 year in the entire group. **(F–I)** Calibration curves and ROC for 1-, 3-, and 5-year in training and testing groups, respectively.

A multivariate and univariate Cox analysis of clinical variables, including age, grade, stage, T stage, and risk scores, revealed that the risk model was the most significant prognostic factor (Figures 4A, B). Then, according to the critical variables in the multiple regression analysis (*p*< 0.05), a prognostic nomogram of bladder cancer patients was established (Figure 4C), which could be used to predict the 1-, 3-, and 5 year OS rates of patients. In the entire cohort, the AUC of values for 1-, 3-, and 5 year OS were 0.783, 0.765, and 0.760, respectively (Figure 4E). Calibration plots for 1-, 3-, and 5 years were generated to verify our model across the entire cohort. All calibration plots fall near the 45-degree diagonal line (Figure 4D). The AUC values and calibration plots of the training set and test set show that the nomogram has good discriminative power (Figures 4F–I).

### GSEA analysis of platinum resistance-related (PRR) LncRNAs

**FIGURE 5:**
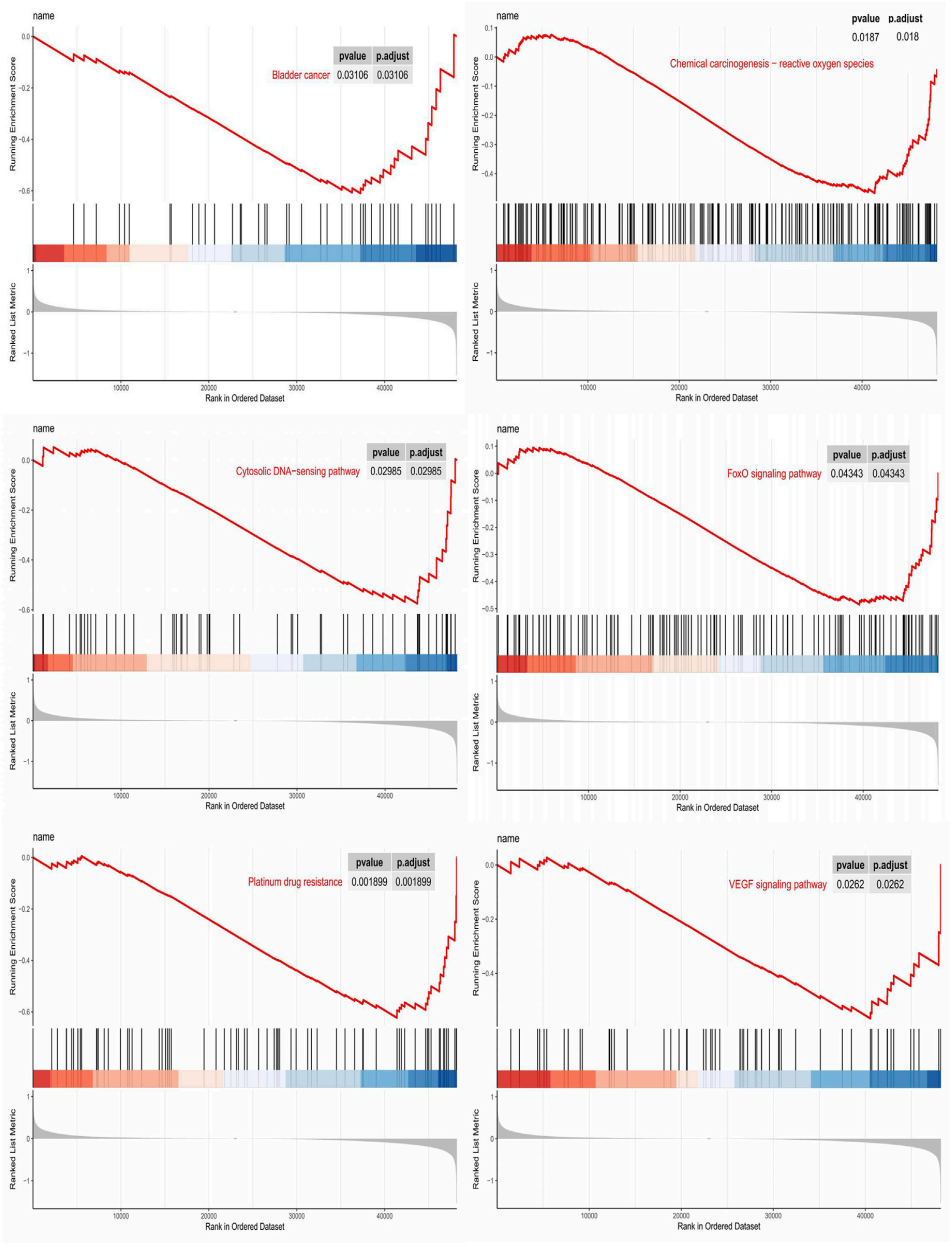
GSEA analysis.

GSEA analysis was performed to elucidate the biological function of PRR-based signatures further. GSEA revealed that PRR lncRNA prognostic models mainly regulated cancer- and platinum-related pathways, such as Bladder cancer, Cytosolic DNA−sensing pathway, VEGF signaling pathway, FoxO signaling pathway, Chemical carcinogenesis−reactive oxygen species, and Platinum drug resistance (Figure 5).

### Immune-related analysis of BLCA patients using the risk model

**FIGURE 6:**
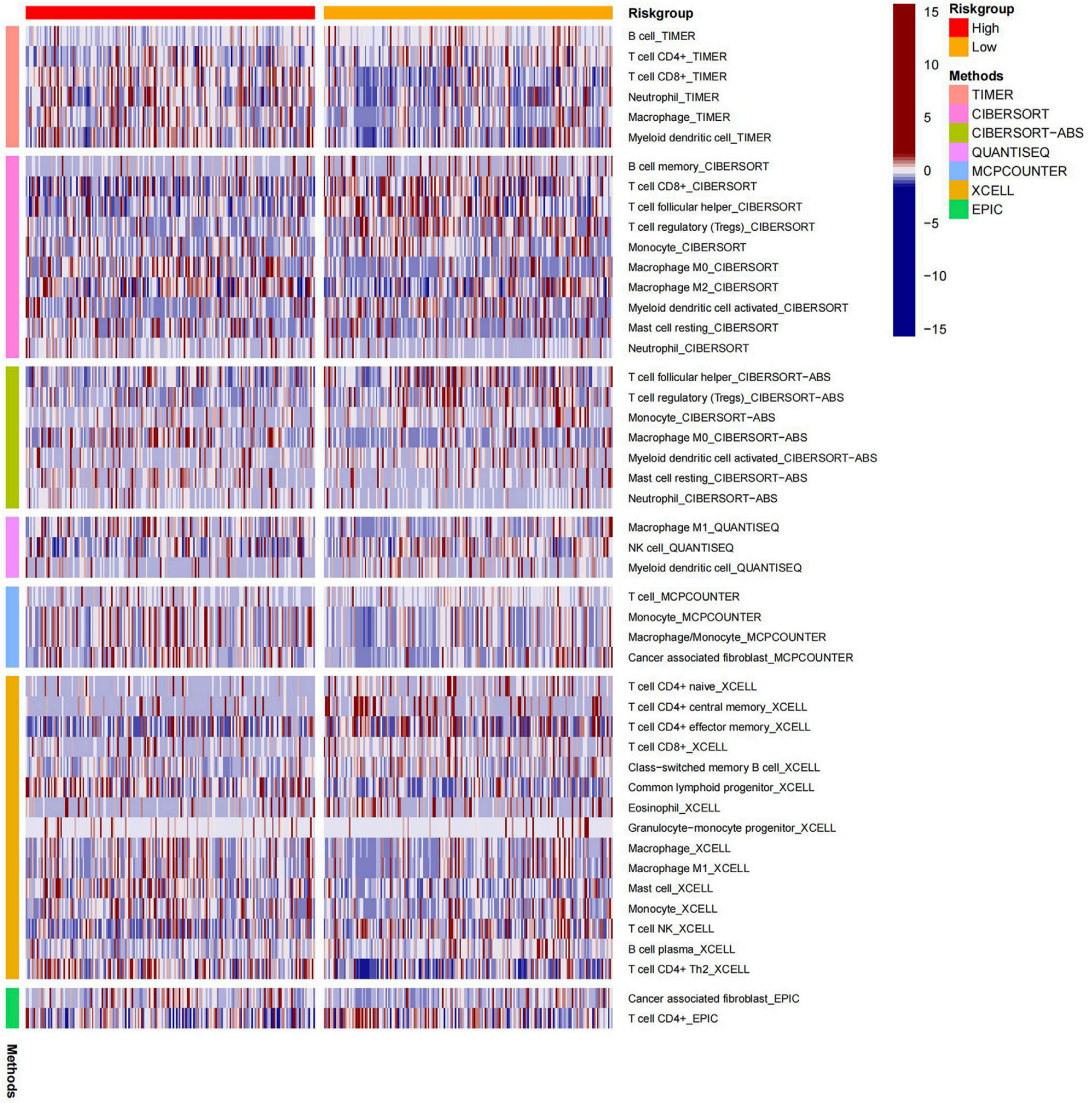
Heat map of immune cell infiltration in high and low-risk groups.

**FIGURE 7:**
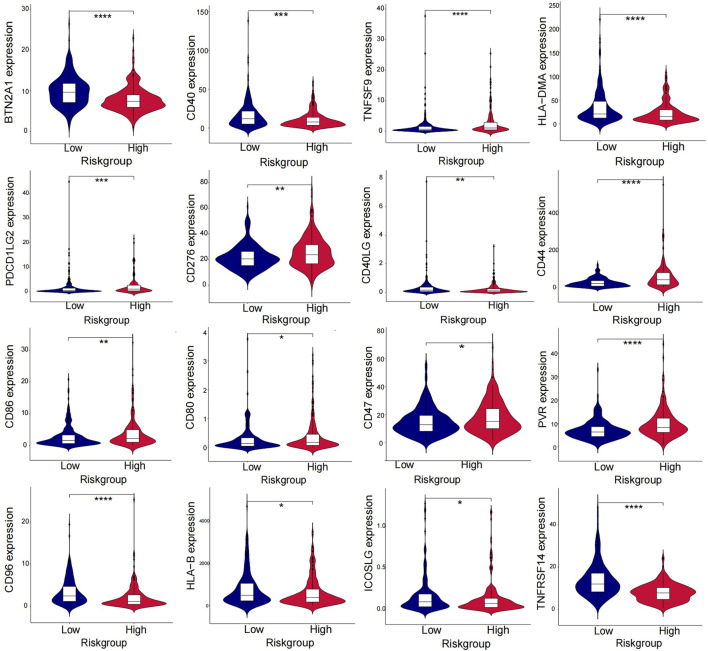
Differences in immune checkpoints between high and low-risk groups.

**FIGURE 8:**
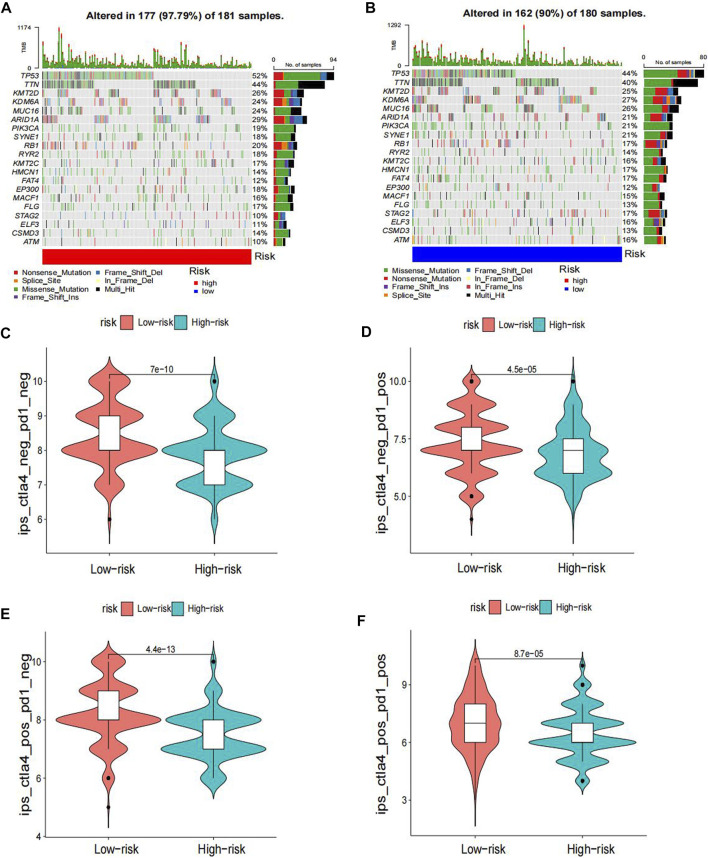
Differences in TMB and immunotherapy sensitivity between the two groups.

The heatmap displayed the relationship between the risk model and immune infiltration (Figure 6). In the low-risk group, CD4^+^ T-cell, CD8^+^ T-cell, and regulatory cells infiltrated more than in the high-risk group. At the same time, macrophages and monocytes were more prevalent in high-risk populations. Furthermore, based on immune checkpoint analysis, representative immune checkpoint-related genes, such as PDCD1LG2, CD44, CD47, CD276, PVR, and TNFSF9, were remarkably upregulated when compared with low-risk group samples (Figure 7). Comparison of somatic mutations in patients with high and low-risk scores and visualization of the top 20 genes with the highest mutation frequency (Figures 8A, B). There was no significant difference in TMB between the high-risk and low-risk groups. By analysis, we found that patients with low-risk scores were more sensitive to immunotherapy, whether they were CTLA4+ or PD-1+ or both positive (Figures 8C–F).

### Drug sensitive and qRT-PCR

**FIGURE 9:**
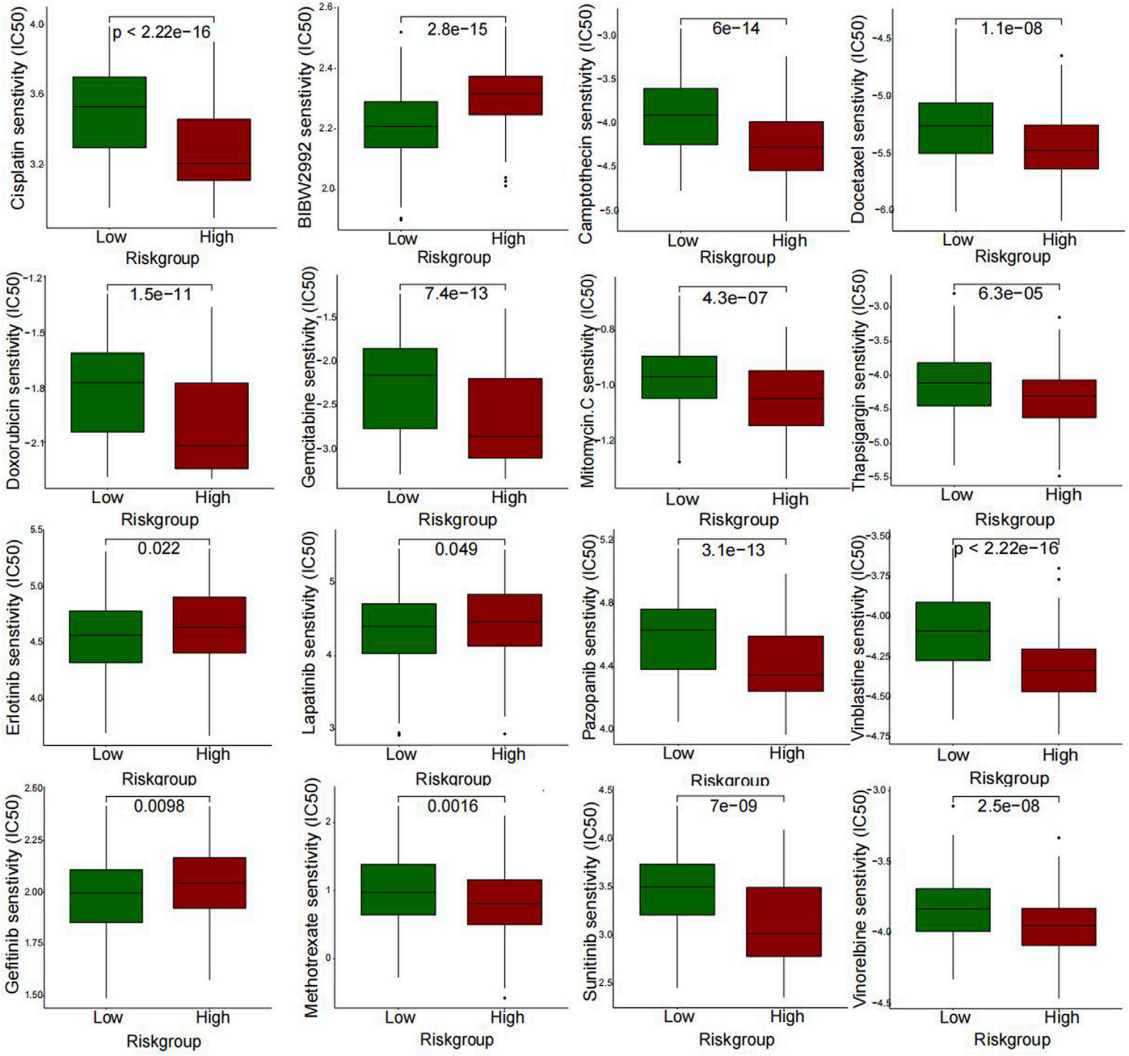
Differences in drug sensitivity between the two groups.

**FIGURE 10:**
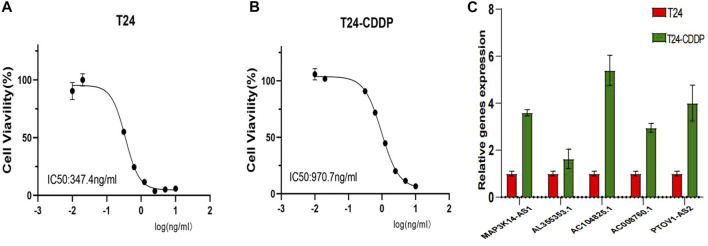
**(A, B)** Cisplatin IC50 for T24-CDDP and T24. **(C)** The expression of AC008760.1, PTOV1-AS2, AL355353.1, AC104825.1, and MAP3K14-AS1 in T24 and T24-CDDP cell lines.

A further investigation was conducted to assess the sensitivity difference of drugs in two groups of patients with bladder cancer to improve the therapeutic outcome. The analysis results indicated that IC50 values of drugs, including BIBW2992, Erlotinib, Gefitinib, and Lapatinib, were higher in high-risk patients than those of low risk. While IC50 values of drugs containing Cisplatin, Gemcitabine, Mitomycin C, Methotrexate, Vinblastine, Vinorelbine, Doxorubicin, Docetaxel, Thapsigargin, and Pazopanib were much higher in the low-risk patients than those of the high-risk (Figure 9). In Figures 10A, B, we can see that the cisplatin IC50 for T24-CDDP was significantly higher than that of T24, implying the thriving culture of our drug-resistant cells. As shown in Figure 10C, AC008760.1, PTOV1-AS2, AL355353.1, AC104825.1, and MAP3K14-AS1 were more highly expressed in cisplatin-resistance T24 cells than normal T24 cells.

## Discussion

In recent years, many studies have focused on the role lncRNAs play in bladder cancer (BLCA). Lia et al. developed and validated an eight-pyroptosis-related lncRNA prognostic model for BLCA (Lia et al., 2022). Luo et al. found that lncRNA RP11-89 facilitates tumorigenesis and ferroptosis resistance in BLCA (Luo et al., 2021). Tong et al. constructed a prognostic epithelial-mesenchymal transition-related lncRNA risk model in BLCA (Tong et al., 2021). Hu et al. discussed the roles and mechanisms of lncRNAs in cisplatin chemoresistance, including changes in cellular uptake or efflux of a drug, apoptosis, autophagy, related signaling pathways, and so on 7). However, studies on the prognosis of platinum resistance-associated (PRR) lncRNAs in BLCA are still limited. Accordingly, we explored the relationship between PRR lncRNAs and the prognosis of BLCA.

Based on eight PRR lncRNAs, we established a BLCA risk prognosis model, and median risk scores categorized patients into high and low-risk groups. Kaplan-Meier survival, heatmap, and ROC analyses have shown the good predictive ability of our risk model. Moreover, the immune-related and drug-sensitivity analysis also showed significant differences between high- and low-risk groups. These identified PRR lncRNAs were protection factors: PSMB8-AS1, AL731567.1, AC104825.1, AC009065.8, MAP3K14-AS1, AL355353.1, AC008760.1, and PTOV1-AS2. When protective factors are expressed at a higher level, the prognosis for BLCA patients is better.

Recent studies have found LncRNA PSMB8-AS1 to be a prognostic marker and a protective factor in BLCA (Tong et al., 2021; Mo et al., 2022). Zhang et al. proposed that PSMB8-AS1 promotes pancreatic cancer progression by regulating the miR-382–3p/STAT1/PD-L1 axis. Thus, it is worthwhile to explore PSMB8-AS1’s mechanism of action in bladder cancer (Zhang et al., 2020). MAP3K14-AS1 was recognized as a highly prevalent and specific methylated locus in colorectal cancer, which can be used to monitor tumor burden dynamics in liquid biopsy under different therapeutic regimens (Barault et al., 2018). Kuang et al. revealed that the necroptosis-related lncRNAs MAP3K14-AS1 and AL731567.1 were considered protective effectors in BLCA (XiaYu et al., 2022). AL355353.1 was found to be associated with glycometabolism in BLCA and affected prognosis (Tang et al., 2022). Liu et al. revealed that PTOV1-AS2 might affect the prognosis of pancreatic cancer through TP53-associated signature (Liu et al., 2021). LncRNA AC008760.1 was identified as expressed lower in bladder urothelial carcinoma cells than in normal urothelial cells (Li et al., 2022), which was consistent with our findings. Moreover, the knockdown of AC008760.1 can significantly promote the proliferation and migration of bladder cancer cells. Furthermore, AC009065.8 and AC104825.1 in BLCA are rarely reported in research, and thus, the specific mechanism is also worthy of further investigation (Chen et al., 2020).

We further compared several clinical variables to assess our risk model’s predictive ability. Three independent prognostic factors were identified: age, stage, and risk score. As previously reported, age and stage are prominent risk factors for multiple tumors, including bladder cancer (Hu et al., 2022; Lu et al., 2022). Further comparison showed that the model’s prediction performance is superior to age and stage, demonstrating its high predictive power. To increase the clinical applicability of the model, we used a nomogram to visualize the survival probability of bladder cancer patients. In the training and testing sets, both ROC and calibration curves showed the good predictive ability of the nomogram.

Immunotherapy has shown promising results in the management of BLCA. In our study, high-risk and low-risk groups were compared regarding the immune checkpoint. Figure 7 displays that TNFSF9, PDCL1LG2, PVR, CD44, CD86, CD80, CD47, and CD276 in the immune checkpoint were expressed in the high-risk group, while BTN2A1, CD40, CD40LG, HLA−DMA, HLA−B, CD96, ICOSLG, and TNFRSF14 were mainly expressed in the low-risk group. For the former, except for TNFSF9, almost all other genes in the immune checkpoint were reported in bladder cancer and were associated with poor outcomes (Kiss et al., 2019; Kucan Brlic et al., 2019; Hu et al., 2020; Yang et al., 2020; Yan et al., 2021; Harland et al., 2022). It is thought that tumors with more mutated genes tend to produce more mutant RNAs and proteins that are more easily recognized by the immune system and respond well to immunotherapy. Thus, we also analyzed the difference in TMB in the two risk groups. Although there was no significant difference in TMB between the high-risk and low-risk groups, we found that the low-risk group gained more immunophenoscores, which can be used to predict response to immune checkpoint inhibitors. The results showed that patients with low-risk scores were more sensitive to immunotherapy, whether they were CTLA4+ or PD-1-. Therefore, combined with our risk model, we found that immunotherapy could be a good option for bladder cancer patients with platinum resistance.

Many studies show that immune infiltration correlates with prognosis (Hatogai and Sweis, 2020; Zheng et al., 2020). Consequently, the rate of immune cell infiltration between different risk groups was calculated. Comparing the low-risk group with the high-risk group, we found that CD8^+^ T-cell and regulatory cells were significantly increased. Induced tumor cell death is the primary function of CD8^+^ cells (Henning et al., 2018). Moreover, the numbers of macrophages and monocytes have risen notably in the high-risk group, which are generally involved in defending against external attacks (Xia et al., 2020). Due to this, we considered that platinum resistance-related lncRNA is closely related to immune infiltration in bladder cancer.

Drug sensitivity analysis showed that high-risk groups were more sensitive to cisplatin because of their relatively low expression of platinum-resistance-associated LncRNAs, which further adds to the reliability of our findings. Moreover, drug analysis results also showed that IC50 of BIBW2992, erlotinib, gefitinib, and lapatinib were lower in low-risk patients, implying that drug-resistant patients were more sensitive to those drugs. T24 bladder cancer cells are inhibited in proliferation and invasion by BIBW2992/Afatinib (Tang et al., 2015). A primary mechanism of gefitinib is that it interferes with the metabolic functions of tumor cells and inhibits EGFR signaling in a meaningful manner (Peng et al., 2016). One recent study suggests lapatinib as a first-line option for treating muscle-invasive urothelial carcinoma in dogs (Maeda et al., 2022). These four drugs all belong to EGFR family inhibitors, which are expected to play a significant role in future bladder cancer treatments. Although we did not find suitable primer sequences for the rest three lncRNAs, it may be because their base sequences are long or technical problems. For the most, we evaluated the expression level of most lncRNAs in our signature. The expression trend followed the bioinformatic prediction.

However, some areas still need to be addressed in this study. Firstly, this is a retrospective study using TCGA datasets. Retrospective studies may have selection and information bias. For example, in light of the small sample size, stage I was grouped with stage II. Secondly, external validation needed to be improved as other databases lacked lncRNA expression profiles or overall survival data. Finally, although we experimentally validated the differential expression of PPR lncRNAs in platinum-resistant bladder cancer cells, the underlying mechanisms of how the detected platinum-resistance-related lncRNAs impact the prognosis of bladder cancer require further study by basic experiments.

## Conclusion

Based on eight PRR lncRNAs, we constructed a prognosis model for BLCA patients. As well as providing prognostic information and immune analysis, our risk model can give a new direction for chemotherapy or targeted therapy for BLCA patients.

## Data Availability

The original contributions presented in the study are included in the article/Supplementary Materials, further inquiries can be directed to the corresponding authors.
