# Supplementary figures and images for: Identification of platinum resistance-related gene signature for prognosis and immune analysis in bladder cancer
Source: Front Genet. 2023 Jan 26;14:1062060. doi: 10.3389/fgene.2023.1062060 (PMC9908994; doi:10.3389/fgene.2023.1062060)

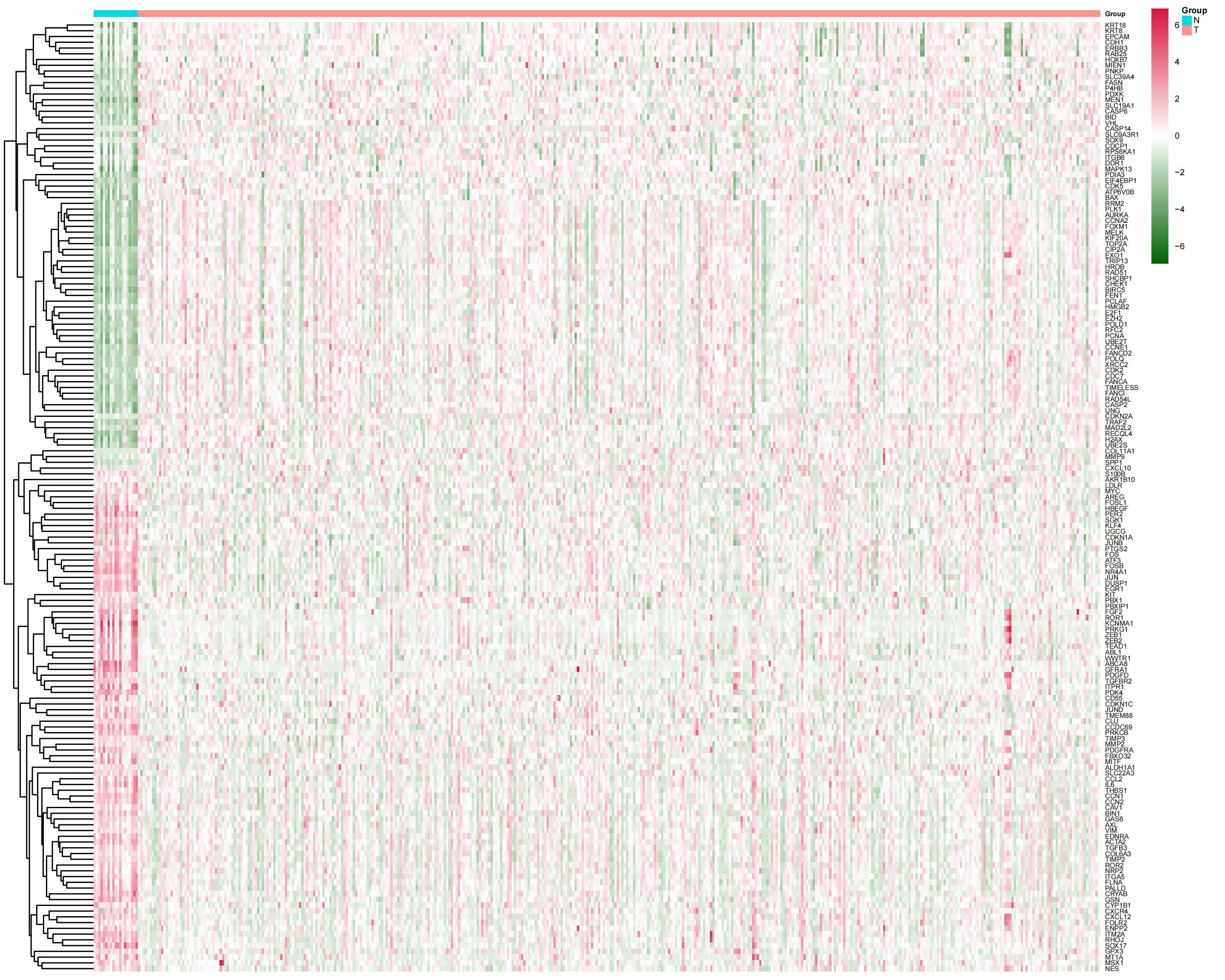

Supplement: Supplementary file 3 [file Image1.JPEG]

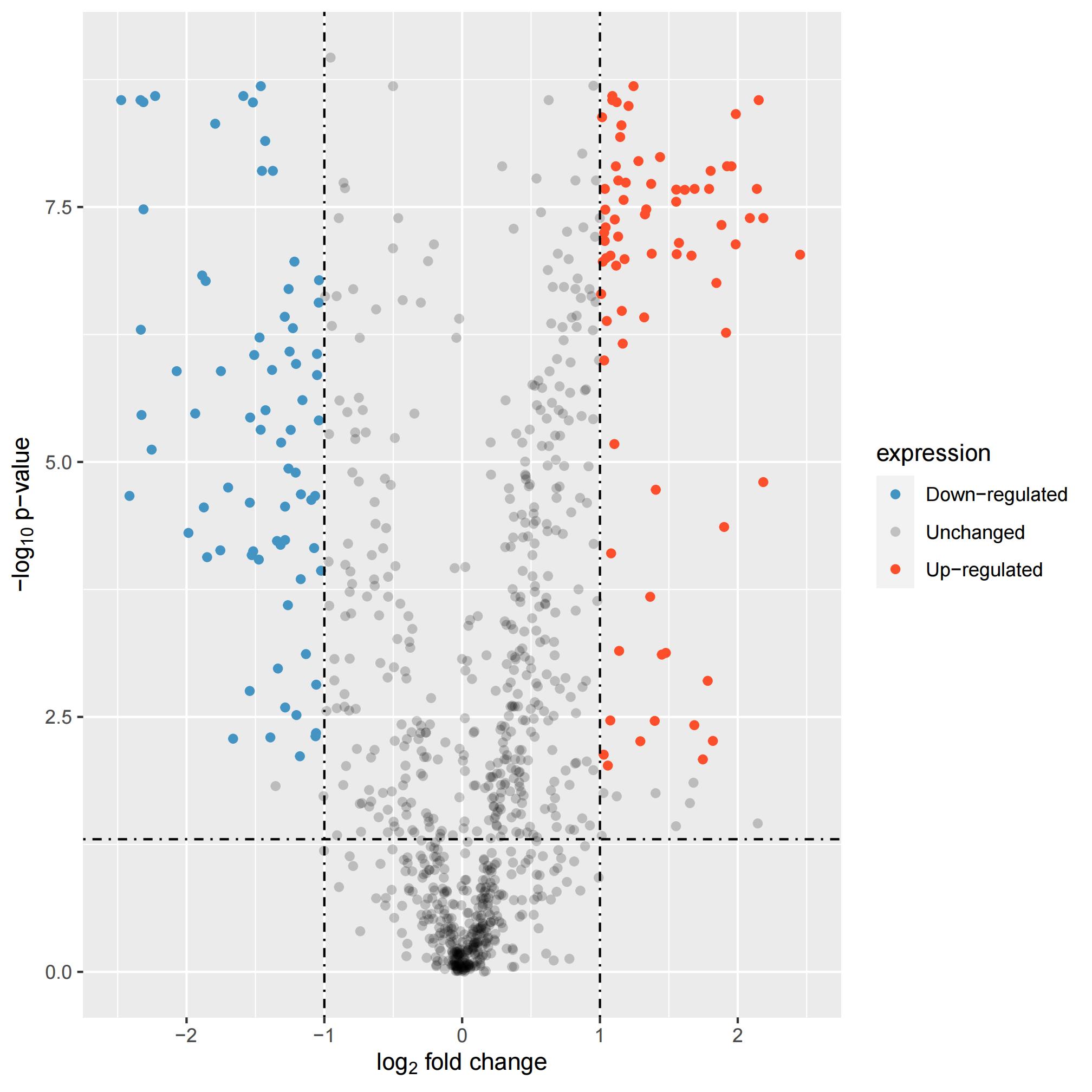

Supplement: Supplementary file 4 [file Image2.JPEG]
