# Supplementary material for: Identification of platinum resistance-related gene signature for prognosis and immune analysis in bladder cancer
Source: Front Genet. 2023 Jan 26;14:1062060. doi: 10.3389/fgene.2023.1062060 (PMC9908994; doi:10.3389/fgene.2023.1062060)
Supplement: Supplementary file 5 [file Table9.DOCX]

All R code and Laboratory-data can be download from this website ：<https://www.jianguoyun.com/p/DVU052sQl_2ACxjU6twEIAA.> JUST FOR PEER REVIEW.

If you have any question about this, please feel free to contract me. E-mail:1484077719@qq.com
